# Validation of Real-time PCR Reference Genes of Muscle Metabolism in Harvested Spiny-Cheek Crayfish (*Faxonius limosus*) Exposed to Seasonal Variation

**DOI:** 10.3390/ani10071140

**Published:** 2020-07-06

**Authors:** Natalia Śmietana, Remigiusz Panicz, Małgorzata Sobczak, Piotr Eljasik, Przemysław Śmietana

**Affiliations:** 1 Department of Meat Sciences, Faculty of Food Sciences and Fisheries, West Pomeranian University of Technology in Szczecin, 4 Kazimierza Królewicza Street, 71-550 Szczecin, Poland; natalia.smietana@zut.edu.pl (N.Ś.); malgorzata.sobczak@zut.edu.pl (M.S.); peljasik@zut.edu.pl (P.E.); 2 University of Szczecin, Institute of Marine and Environmental Sciences, 18 Adama Mickiewicza Street, 70-383 Szczecin, Poland; przemyslaw.smietana@usz.edu.pl

**Keywords:** ferritin, endogenous control genes, freshwater crayfish, molting, abdomen muscles, troponin c

## Abstract

**Simple Summary:**

In the age of shrinking stocks and intensive animal production alternative raw materials are in the eye of the scientists. Here, we aimed to identify molecular tools to evaluate the quality of spiny-cheek crayfish meat. Results showed a set of genes that were steadily or deferentially expressed between seasons and sexes. Additionally, the authors revealed genes involved in molting cycle and muscle growth/atrophy of the crayfish. The suite of molecular tools developed in the study have potential for the profiling of crayfish raw material quality in the food sector.

**Abstract:**

Real-time quantitative reverse transcription PCR (RT-qPCR) is a sensitive and broadly used technique of assessing gene activity. To obtain a reliable result, stably expressed reference genes are essential for normalization of transcripts in various samples. To our knowledge, this is the first systematic analysis of reference genes for normalization of RT-qPCR data in spiny-cheek crayfish (*Faxonius limosus*). In this study, expression of five candidate reference genes (*actb*, *β-actin*; *gapdh*, glyceraldehyde-3-phosphate dehydrogenase; *eif*, eukaryotic translation initiation factor 5a; *ef-1α,* elongation factor-1α; and *tub*, α-tubulin) in muscle samples from male and female F. limosus in spring and autumn was analyzed. Additionally, the most stable reference genes were used for accurate normalization of five target genes, i.e., *tnnc,* troponin c; *ak*, arginine kinase; *fr*, ferritin; *ccbp-23*, crustacean calcium-binding protein 23; and *actinsk8,* skeletal muscle actin 8. Results obtained using the geNorm and NormFinder algorithms showed high consistency, and differences in the activity of the selected *actb* with *eif* genes were successfully identified. The spring and autumn activities of the target genes (except *ak*) in the muscle tissue of males and females differed significantly, showing that both sexes are immensely involved in an array of breeding behaviors in spring, and females intensively recover in the autumn season. Characterization of first reference genes in spiny-cheek crayfish will facilitate more accurate and reliable expression studies in this key species.

## 1. Introduction

The food sector usually assess quality parameters of raw material by means of basic chemical analyses (e.g., protein and mineral content, composition of fatty acids) but also based on processing (shear force value, drip loss) and sensory parameters (texture, juiciness) [1]. However, novel analytic approaches employ molecular methods to provide information about changes occurring at the cellular level [2], which affect raw material quality. Such analyses can employ the commonly used technique of real-time PCR, which allows for measuring of the activity of genes encoding proteins involved in key metabolic pathways, including development and function of muscles, and ultimately affecting the quality of meat [3,4,5]. Decker and Welch [6] showed that increased concentration of the protein ferritin causes active binding of iron, which in turn leads to a reduction in meat quality due to an increased level of fat oxidation. Other examples of genes and their protein products with an impact on muscle function include arginine kinase, which regulates energy generation [7], as well as troponin c and actin, which enable muscle contraction in common shore crab (*Carcinus maenasi*) [8,9]. As for the latter pair of genes, it has been demonstrated that low water temperature in autumn and winter reduces their expression, which in turn results in poorer growth of the muscle tissue. Genes associated with muscle function have been mostly identified to date in farm animals [10] and fish [11] while to a low extent in invertebrates, like crayfish. Moreover, for the latter group of animals a set of suitable reference genes have not been characterized that could potentially be used in other studies, such as environmental, epidemiological or behavioral.

Spiny-cheek crayfish (*Faxonius limosus*, Rafinesque, 1817) is a freshwater species, probably the most common invasive member of the Astacoidea superfamily in Central Europe, certainly in Poland. Since the end of the 19th century, when only a hundred individuals were imported from North America to Europe by the German breeder Max von dem Borne [12], the species had not only spread over almost the entirety of Poland, but also started prevailing over native species, especially noble crayfish (*Astacus astacus*). The ecological success of the species was possible primarily thanks to its survival strategy determined by such features of the population such as high fertility, early reproductive maturity and resistance to changing environmental conditions [13]. Moreover, the species is a vector for a water mold *Aphanomyces astaci*—the causative factor of a crustacean disease called crayfish plague which leads to an epizootic among native crayfish, mainly noble crayfish. One of the causes of the success of the invasive spiny-cheek crayfish was the fact that catches of noble crayfish had decreased significantly. However, the commercial value of *F. limosus* scaled to reflect its edible parts is 24% and is comparable with that of the highly appreciated *A. astacus* [14]. Depending on the season and production-related characteristics of the reservoir, catches of spiny-cheek crayfish can have a relatively high efficiency, between 1.5 and 10 kg per hectare per year (Śmietana, data unpublished). Compared with the average fishery performance for Polish lakes, amounting to 25–40 kg of fish per hectare, the above value can be considered interesting from an economic perspective. The constant increase of the freshwater *F. limnosus* population in Europe has attracted the attention of the food and catering industry due to the various profit opportunities. Study results published to date have indicated two main routes of exploitation of crayfish, i.e., as a source of edible and non-edible material. The former mainly includes muscles of the abdomen and, to a lesser extent, claws. Crayfish meat is characterized by a high protein content of 18–20% and a low-fat content of 0.8–2.8%. This material contains 13.7–16.2% of polyunsaturated omega-3 fatty acids and 19–23% of polyunsaturated omega-6 fatty acids [15]. The crayfish processing industry also provides a significant quantity of by-products, with astaxanthin being known as an aquafeed antioxidant for salmonids [16] or chitosan, produced from chitin, which is highly valued by the pharmaceutical, cosmetic and food industries [17].

Detailed characterization of the reference and muscle-specific genes in that species is a key element in the potential commencement of economic exploitation of spiny-cheek crayfish by the food and catering industry. It is an important issue especially in light of the fact that conditions in the colonized aquatic environment play an important role in shaping the characteristics of the muscle tissue. Therefore, the aim of this study was to characterize and assess the level of activity of selected reference genes, i.e., β-actin *(actb*), glyceraldehyde-3-phosphate dehydrogenase (*gapdh)*, eukaryotic translation initiation factor 5a (*eif*), elongation factor-1α (*ef-1α*) and α-tubulin (*tub*). Moreover, assessment included the activity of five genes encoding proteins involved in muscle metabolism, i.e., arginine kinase (*ak*), ferritin (*fr*), crustacean calcium-binding protein 23 (*ccbp-23*), troponin c (*tnnc*) and skeletal muscle actin 8 (*actinsk8*), depending on the fishing season (spring vs. winter) and crayfish sex (females vs. males).

## 2. Materials and Methods

### 2.1. Animals and Sampling 

The study was accepted by the Faculty Board under the number 517-08-026-7724/17 and no restrictions were raised by the Ethics Committee. Three-year-old spiny-cheek crayfish of each sex were obtained in the spring (May) and autumn (September) of 2017 by free diving in Lake Sominko (latitude 54^○^04’46’’N; longitude 17^○^52’48’’E) located on the Charzykowy Plain in the area surrounding (the buffer zone) the Wdzydze Landscape Park. Immediately after catching, the crayfish were stored in a water-filled container for transport to the laboratory, where they were stored at 4 °C. Afterwards, five females and five males of crayfish per batch (spring, autumn) were randomly collected, euthanized and dissected. Muscle samples (~50 mg) were collected from the abdomen, placed in 2-mL tubes containing 500 μL of TRIzol^®^ Reagent (Sigma-Aldrich, Hamburg, Germany) and 20 zirconium oxide beads (2.6–3.3 mm), homogenized for 30 s at 5000 rpm using a Minilys personal homogenizer (Bertin Technologies, Aix-en-Provence, France), and stored at −80 °C until analysis.

### 2.2. Total RNA Isolation and cDNA Synthesis

Total RNA from each sample was extracted using the Direct-zol™ RNA MiniPrep kit (Zymo Research, Irvine, CA, USA) and treated with DNase I to avoid genomic DNA contamination, in accordance with the kit instructions. The purified RNA was dissolved in 15 µl of nuclease-free water (Sigma-Aldrich, Hamburg, Germany). The RNA concentration and purity were quantified using a NanoDrop 2000 spectrophotometer (Thermo Fisher Scientific, Dreieich, Germany). The integrity of the purified RNA samples were examined by 1.5% (p/v) agarose gel electrophoresis. The samples at absorption ratios of A260/A280 = 1.9–2.1 and A260/A230 ≥ 2.0 were used for cDNA synthesis. First-strand cDNA was synthesized with the High-Capacity cDNA Reverse Transcription Kit (Thermo Fisher Scientific, Dreieich, Germany) following the kit instructions, using 0.5 µg of RNA and 1 μg of oligo-dT in a final volume of 20 μL.

### 2.3. Identification and Characterization of Candidate and Target Genes

The candidate genes selected for the study served as reference genes that had been previously reported as suitable for gene expression normalization in red swamp crawfish (*Procambarus clarkii)* in different experimental conditions [4]. Five potential reference genes were selected for expression stability analysis, namely β-actin (*actb*), glyceraldehyde-3-phosphate dehydrogenase (*gapdh*), eukaryotic translation initiation factor 5a *(eif*), elongation factor-1α (*ef-1α*) and α-tubulin (*tub*). Moreover, five target genes were selected based on expression profiles obtained for the muscle of red swamp crawfish [18], namely skeletal muscle actin 8 (*actinsk8*), troponin c (*tnnc*), crustacean calcium-binding protein 23 (*ccbp-23*), ferritin (*fr*) and arginine kinase (*ak*) (Table 1). To ensure the reliability and correctness of the proposed reference and target genes, we first performed gradient polymerase chain reaction (PCR) to establish annealing temperatures for the selected primers using synthetized cDNA as template. Briefly, PCR was performed using a MasterCycler 5331 gradient (Eppendorf, Hamburg, Germany) and prepared using the GoTaq® G2 Flexi DNA Polymerase kit (Promega, Walldorf, Germany) comprising 1× PCR buffer, 200 μM of dNTPs (Thermo Fisher Scientific), 2.5 mM of MgCl2 (25 mM), 0.5 U of Taq polymerase, 0.5 μM of each primer (Genomed, Warsaw, Poland), 1 μL of DNA template, topped up with milli-Q water to 25 μL. Samples were initially denatured at 94 °C for 5 min, followed by 35 cycles of 30 s denaturation at 94 °C, 30 s annealing at 50–65 °C, and 30 s extension at 72 °C, and followed by final extension at 72 °C for 7 min. The results of DNA extraction and amplification were assessed by separating the samples on 2% agarose gel. Subsequently, conventional PCR was performed for each of the selected genes, using the established annealing temperatures according to the conditions described above. The obtained PCR products were bidirectionally sequenced by Genomed (Warsaw, Poland) and assembled with Geneious 8.1.6 [19]. Finally, all sequences were searched for matches against the GeneBank database using BLASTn to confirm their identity. 

### 2.4. Phylogenetic Analysis of the Gapdh Gene

The *gapdh* gene sequence was selected for phylogenetic analysis because it was the most informative across investigated candidate genes. The deduced amino acid (aa) sequences of putative gapdh proteins isolated from *F. limosus* and the functionally characterized gapdh obtained from NCBI were aligned using MAFFT v7.18 with the E-INS-i strategy [20]. Cleaned alignments were subjected to a maximum likelihood phylogenetic analysis using RAxML with 1000 rapid bootstrap replicates. The best-fit evolutionary model was selected to LG + G + I for both genes by MEGAX [21]. The resultant RAxML trees were visualized using Interactive Tree of Life v3 [22].

### 2.5. Quantitative Real-Time PCR

Amplification of the reference and target genes was performed on a LightCycler® 96 Instrument (Roche Life Science, Penzberg, Germany). Individual real-time PCRs were carried out in a final reaction volume of 10 µl, using 5 µl of GoTaq® qPCR Master Mix (Promega, Walldorf, Germany), 3 µl of ultrapure water (Sigma-Aldrich, Hamburg, Germany), 0.5 µl of each specific primer (10 µM, Table 1) and 1 µl of 5× diluted cDNA template. Each transcript was amplified, using technical triplicates per sample, under the following conditions: 95 °C for 2 min, followed by 40 cycles of a two-step amplification program (15 s at 95 °C; 1 min at 60 °C), according to the primer set used (Table 1). A melting curve analysis (60–95 °C) was performed to verify that only specific amplification occurred, and no primer dimers were amplified. Negative controls with RNA were run to ensure no genomic DNA amplification. The data were obtained as Ct. Correlation coefficient (R2) was determined based on the slopes of the standard curves generated with a ten-fold dilution series of sample cDNA from *F. limosus* muscle RNA. R2 values above 0.98 were accepted. qRT-PCR efficiency (E) was determined using the equation: E = 10(−1/slope) [23]. No amplification product was observed in negative controls and no primer–dimer formations were observed in the templates.

### 2.6. Algorithms for the Selection of Reference Genes

To identify the most appropriate reference genes based on the calculated expression stability values, two algorithms were used: geNorm [24] and NormFinder [25]. According to the rankings generated by the algorithms, an appropriate weight was assigned to each gene and a geometric mean of their weights was calculated for the overall final ranking. Two reference genes characterized by the lowest variability of expression levels in the tested samples (muscle samples from the abdomen of females and males from two seasons) were chosen as the internal control used to assess the expression of the spiny-cheek crayfish target genes.

### 2.7. Gene Expression Profiling

Activities of the five target genes, i.e., *actinsk8, tn1nc, ccbp-23, fr* and *ak* in muscle samples from the abdomens of *F. limosus* females and males collected during spring and autumn seasons were normalized using selected reference genes. qPCR was performed as described above using primer pairs listed in Table 1. Relative expression of the target genes in the *F. limosus* samples were calculated in the GeneEx software (MultiD Analyzes AB, Gothenburg, Sweden) using the 2**^-ΔΔCt^** method [26]. Results were further analyzed using the Statistica 13 software (StatSoft Inc., Cracow, Poland) to determine significant differences at *p* ≤ 0.05 and *p* ≤ 0.01 with one-way ANOVA followed by Tukey HSD post-hoc test. 

## 3. Results and Discussion

### 3.1. Sequences and Phylogenetic Analysis of the Candidate and Target Genes 

**Figure 1:**
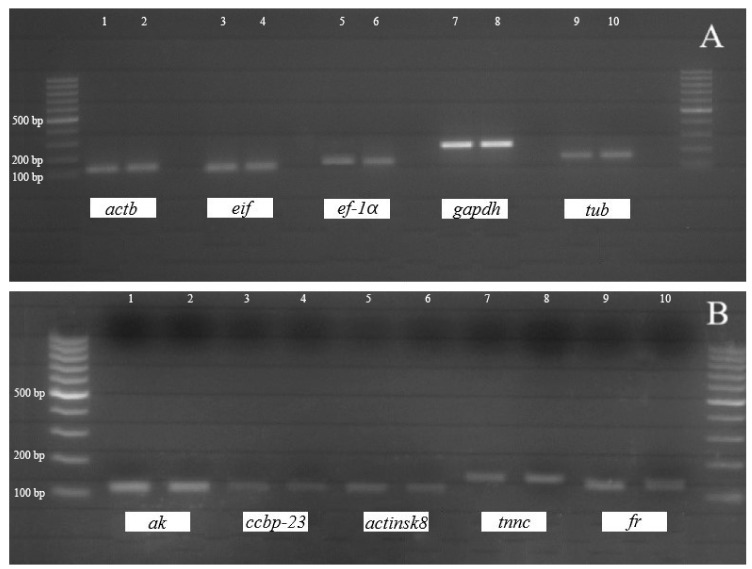
Electropherogram of the qPCR products of the five candidate reference (**A**) and target (**B**) genes in the muscle tissue of *F. limosus*. Abbreviations: *actb*, β-actin; *gapdh*, glyceraldehyde-3-phosphate dehydrogenase; *eif*, eukaryotic translation initiation factor 5a; *ef-1α*, elongation factor-1α; *tub*, α-tubulin; *tnnc*, troponin c; *ak*, arginine kinase; *fr*, ferritin; *ccbp-23*, crustacean calcium-binding protein 23; *actinsk8*, skeletal muscle actin 8. Molecular weight 100–1000 bp.

**Figure 2:**
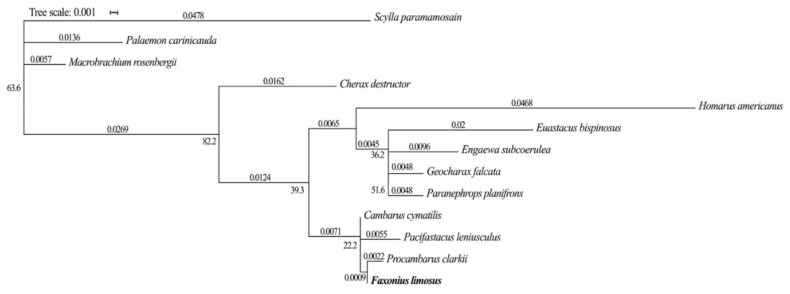
The phylogenetic tree for the *gapdh* gene of *F. limosus***.** Abbreviations: figures above the branches and next to the nodes denote their length and bootstrap value, respectively.

Analysis of the PCR products on agarose gel (Figure 1) and BLASTn of all obtained partial sequences of the candidate reference and target genes against the NCBI nucleotide database confirmed the origin of the sequences. All assembled partial nucleotide sequences of the candidate reference and target genes were deposited in GenBank under the following names and numbers: eukaryotic translation initiation factor 5a (MN958081), elongation factor-1α (MN958082), α-tubulin (MN958083), glyceraldehyde-3-phosphate dehydrogenase (MN958084), β-actin (MN958085), arginine kinase (MN958080), ferritin (MK246411), crustacean calcium-binding protein 23 (MK246412), troponin c (MK246413) and skeletal muscle actin 8 (MK246414). The phylogenetic tree for the *gapdh* gene of *F. limosus* showed a close clustering with the *gapdh* gene from the Cambaridae (*P. clarkii*, *Cambarus cymatilis*) and Astacidae (*Pacifastacus leniusculus*) families (Figure 2). The phylogenies of the freshwater crayfish subfamily Cambarinae using partial data from the 16S ribosomal RNA gene published by Johnson et al. [27] were significantly similar to that obtained in our study, even despite reclassification of spiny-cheek crayfish from the *Orconectes* genus to the *Faxonius* genus [28]. Therefore, the resulting maximum likelihood clustering could confirm the expected putative functionality of the investigated genes.

### 3.2. Stability and Ranking of Reference Genes in F. limosus 

**Figure 3:**
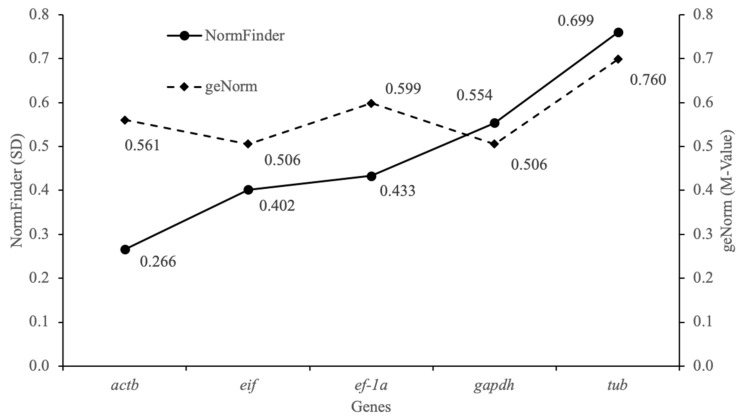
Ranking of gene stability values provided by geNorm and NormFinder. Five potential reference genes were tested for stability in the muscles of *F. limosus* females and males in spring and autumn.

Since the stability of a reference gene is variable, genes with the most stable expression for one comparison (e.g., target genes in the abdomen of female vs. male from spring) can be highly variable in another (e.g. target genes in the abdomen of female vs. male from autumn). In general, the lower the M (geNorm) and SD (NormFinder) values are, the more stable expression of the candidate gene is. Therefore, we analyzed the data based on individual comparisons to search for pairs of the best reference genes for each comparison. The results showed that among the different seasons and sexes, *actb* and *eif* had the most stable expression for all samples (Figure 3), as well as for each tested comparison (Table 2). Although *actb* is one of the most commonly used reference genes in numerous decapods, e.g., peppermint shrimp, *Lysmata wurdemanni* [29]; whiteleg shrimp, *Litopenaeus vannamei* [30]; claw crayfish, *Cherax quadricarinatus* [31]; red swamp crayfish *P. clarkii* [32], its expression may vary considerably between the nine analyzed tissues (heart, muscle, ganglion, intestine, stomach, liver, kidney, gill and gonad) and/or samples (control vs. molting stages) as shown for narrow-clawed crayfish, *Astacus leptodactylus* [33]. Validation of the usefulness of the selected reference genes in *P. clarkii* demonstrated that *eif* and *18S* (18S ribosomal RNA) were the optimal reference genes for expression data from different tissues [4]. Similar results were obtained by Nicosia et al. [34], who reported that the *18S rRNA* and *eif* genes showed stable expression levels in the same species to analyze differences in the activity of the prophenoloxidase (*proPO*) gene in *P. clarkii*. In the presented study, *gapdh* and *tub* were the least stably expressed, while *ef-1α* exhibited an intermediate level of activity among the analyzed candidate reference genes. As reported by Jiang et al. [4], *ef-1α* was the least stably expressed gene, and the authors showed that this gene should not be used as a reference for normalization of qRT-PCR data in red swamp crayfish, despite being an ideal reference gene in the ovarian tissue from giant tiger prawn, *Penaeus monodon* [35]. In the case of *gapdh* and *tub*, low expression stability was also observed in different tissues of *P. clarkii* [4], marbled crayfish, *Procambarus virginalis* [36], and oriental river prawn, *Macrobrachium nipponense* [37]. However, in the same species, i.e., red claw crayfish, *gapdh* was successfully used to normalize pre- and post-WSSV infection gene expression data in crayfish Hpt cell cultures [38]. To date, no studies of suitable reference genes in *F. limosus* have been reported among the increasing number of studies focused on identifying stably expressed genes in other crustaceans [4,38]. Here, we characterized and validated first five potential reference genes, and it should be emphasized that no potential reference genes are likely to be stably expressed under all possible experimental conditions. Therefore, it is highly advisable to evaluate stability of further sets of reference genes for gene expression analysis by RT-qPCR in spiny-cheek crayfish under different conditions. 

### 3.3. Quantitative Analysis of ak, tnnc, ccbp-23, fr and actinsk8 in the Abdomen Muscles of Females and Males of F. limosus from Two Seasons

**Figure 4:**
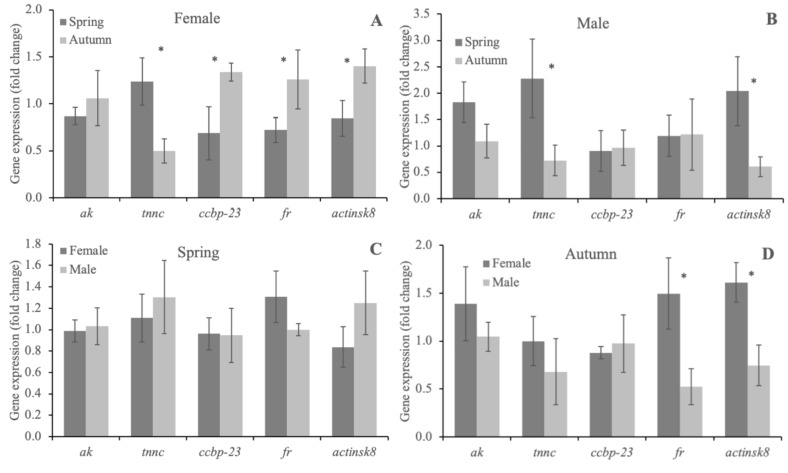
Expression (mean + SD) of *ak*, *tnnc*, *ccbp-23*, *fr*, *actinsk8* in the muscle tissue of *F. limosus* males and females in spring and summer. Abbreviations: **A**—gene expression in female samples in spring and autumn, **B**—gene expression in male samples in spring and autumn, **C**—gene expression in female vs. male samples in spring, **D**—gene expression in female vs. male samples in autumn, *p ≤ 0.05.

Comparison of the activity of the five genes in the abdomen muscles of females and males of *F. limosus* collected during spring and autumn revealed numerous differences that were undoubtedly related to the different timing of activities related to the role of both sexes in the reproduction cycle (Figure 4A–D). In the spring season (reproduction time), both sexes of spiny-cheek crayfish expressed a higher activity of the *tnnc* gene which codes for troponin C (Figure 4A,B). The protein which, together with troponin I and T, is part of a heterotrimeric protein complex (troponin) that decodes muscle Ca*^2+^* signals and induces muscle cell contraction by regulating the interaction between actin and myosin [39]. Therefore, the increased activity of *tnnc* serves as a molecular-level gauge which might reflect the higher activity of myofibrils in the F. limosus abdomen related with an array of female and male reproduction behaviors, especially mating behavior, pleopodal egg care and shelter burrowing [13]. Numerous environmental (water hydrochemistry, quality and quantity of food) and internal (hormones, transcription factors, molting) factors stimulate troponin c transcription and translation which subsequently undergo a differential repression according to the actual state (hypertrophy, atrophy) of the specific muscle type [40]. For example, Chao et al. [41] characterized eleven troponin c isoforms in American lobster (*Homarus americanus*) that were expressed primarily in skeletal muscles, but also in the heart and other tissues, e.g., midgut, hepatopancreas, thoracic ganglion, gill and ovary. BLAST search (unpublished) revealed that the sequence of the *tnnc* gene from this study showed the highest similarity to the *TnC2b* isoform that was expressed in the deep abdominal muscle of the American lobster in which fast glycolytic fibers are located. This phasic type of muscle fibers has a higher (comparing to tonic, slow-twitch oxidative) expression of myosin heavy chain and polyneuronal innervation of several motor axons, and generate spikes and fast twitch contractions when stimulated, e.g., in powerful bursts [42,43]. Interestingly, the study did not reveal differences in the *tnnc* expression when samples of both sexes were analyzed for each season separately (Figure 4C, D). This may indicate a balance in the turnover and functioning of troponin c in both sexes, which may be regulated mainly by water parameters in the lake. Carter and Fraser [44] underlined that water temperature is one of the main factors which influenced contraction rate in a similar way in European green crab (*Carcinus maenas*) and harbor crab (*Liocarcinus depurator*). 

Apart from troponin c, structural proteins such as actin analyzed in this study have a key role in muscle contraction. Expression of the actinsk8 gene confirmed the results obtained for *tnnc* in males from the spring season, but also revealed that the activity of this gene was lower in females collected in autumn (Figure 4A,B). This discrepancy might be explained by the described relationship between stages of the molting cycle and muscle atrophy/growth peaks. Males of the spiny-cheek crayfish grow considerably faster than females, especially in nutrient-rich niches, due to usually two molting events (late spring and summer) in a year, while females, due to necessity of pleopodal egg bearing, undergo a single molting event in July–August. Successful molting must be preceded by a reduction of muscle mass to facilitate removal of old exoskeleton, after which the greatest increase in the body mass is observed [13,45]. Medler et al. [46] showed that synthesis of structural proteins in the muscles of American lobster was significantly increased immediately before and after molting, which is also consistent with our findings of the upregulation of the actinsk8 gene in both sexes. In turn, different expression of the actinsk8 gene between males and females in autumn confirmed the above-mentioned results, since females grow intensively, recover after mating and molting and prepare for the next breeding season in this period. Additionally, high muscle growth of females during the intermolt stage might be partly confirmed by the higher *ccbp-23* gene expression in females collected in autumn compared with those collected in spring (Figure 4D). Sauter et al. [47] characterized *ccbp-23* in the abdomen muscles of *F.* (*Orconencetes*) *limous* and *H. americanus*, described regulatory properties of this protein and evidenced a high similarity of the *ccbp-23* sequence to calcyphosine and to other regulatory calcium sensors, such as calmodulin and troponin c. Lack of differences (Figure 4C) between the recorded activities of the ccbp-23 gene in the spring season, similarly to *tnnc* and *actinsk8*, may have resulted from the fact that both sexes were immensely involved in the energy-demanding mating and spawning behaviors [48]. Further studies are necessary to elucidate the molecular mechanisms behind the differential expression of Ca*^2+^* regulatory proteins *tnnc* and *ccbp-23* in the spring and autumn (Figure 4A,B). 

Discrepancies between sex-linked gene expressions have always been difficult to clarify and confirm, especially in studies in which environmental conditions were defined as key elements. In our study, for the differential expression of the ferritin gene we found further potential evidence that the females of spiny-cheek crayfish in autumn were sampled during their recovery period. Ferritin molecules store iron and play numerous important roles in the body, e.g., regulate cellular homeostasis [49] and are suggested to be involved in cell proliferation [50]. Therefore, the elevated expression of the multi-task *fr* gene in female samples compared with male samples (Figure 4A), as well as in female samples collected in autumn but not in spring (Figure 4D), might have been a signature of enlargement of muscle fibers to the maximum intermolt size. Intensive growth during the intermolt stage has been observed in the muscles of lobster [51], but also crayfish [52], crab [53] and shrimp [54]. However, future work is needed to determine the role of ferritin in the growth and development of the muscle tissue in spiny-cheek crayfish. Interestingly, the expression of the arginine kinase gene was comparable irrespective of sex or season (Figure 4A–D). However, the identified differences in the activity of the *ak* gene were insignificant, and the *ak* expression pattern resembled that of actinsk8. Arginine kinase plays an important role in the physiological function of muscles, osmotic balance, growth and energy metabolism, but also in mechanisms involved in environmental adaptation [55]. In a study assessing the *ak* expression in cuttlefish (*Sepia pharaonis*) under different salinities, it was shown that energy metabolism reactions regulated by this gene may have important effects in low salinity. Additionally, the authors showed that the abundant expression and increased activity of *ak* was correlated with a higher level of protein synthesis in muscles [56]. Another study involving transcriptome profiling in abalone (*Haliotis diversicolor*) showed a harmonized expression of the *ak* and actin genes, but also troponin I–another gene involved in the regulation and contraction of muscles [57]. Although our study did not show any differential expression of the *ak* gene, the results suggest that this gene is involved in energy balance in the muscle tissue of male and female *F. limosus* and has a direct role in the regulation and control of behavior in this species.

## 4. Conclusions

In conclusion, a preliminary validation of first candidate reference genes and an activity analysis of five genes involved in muscle growth and development in males and females of *F. limosus* collected in the spring and summer season were performed in this study. A total of five partial cDNAs encoding five candidate reference (*actb, gapdh, eif, ef-1α, tub*) and five target genes (*tnnc, actinsk8, ccbp-23, fr, ak*) were sequenced. Among the candidate reference genes, the highest stability in the muscle tissue was observed for *actb* and *eif*, and these genes were later used in normalization of gene expression of the target genes. Additionally, the results provided a first insight into the activity of target genes (except *ak*) of females and males in which a differential response was observed in the spring and autumn. Moreover, the set of reference genes identified for the first time in this study will help other research teams to achieve accurate and reliable qPCR results in a wide variety of studies involving spiny-cheek crayfish.

## Figures and Tables

**Table 1:** List of genes analysed in the study including primer sequences.

Gene Name (Symbol)	Cellular Function	Primer Sequence (5′ → 3′)	Product Size(bp)	Reference
α-tubulin (*α-tub*)	Cytoskeletal structural protein	F: CCACTTCCCTCTCGTCACC R: AACAGCACGCCATGTACTTT	155	[4]
β-actin (*actb*)	Cytoskeletal structural protein	F: ATTGCAGACAGGATGCAGAA R: GAAAGGGAAGCCAAGATGG	125	
Elongation factor 1-α (*eif-1a*)	Protein biosynthesis	F: CCACAAAGGCAGGTGAAAAGG R: ATTGGGTGAACCAAGCAGGG	110	
Eukaryotic translation initiation factor 5a (*eif*)	Protein synthesis	F: GGAATAAGGGGACGAAGACC R: GCAAACACACGCTGGGAT	126	
Glyceraldehyde-3-phosphate dehydrogenase (*gapdh*)	Oxidoreductase in glycolysis and gluconeogenesis	F: GCCCAGAACATCATCCCATCT R: CGTCATCCTCAGTGTAACCCAAG	235	
Arginine kinase (*ak*)	High-energy phosphate transfer	F: GGAGTGAGTCAGTGTGAGGC R: GGAGTAGCGTTACCCACGAG	114	[18]
Crustacean calcium-binding protein 23 (*ccbp-23*)	Regulatory protein	F: GGCCATCGATGAAGACGAGT R: TCCGGATCAGCAGTAGTGGA	117	
Ferritin (*fr*)	Iron binding	F: GCTCTTCCTGGGGCATCAAA R: CAGTGTTGGTGCTGCAATGG	132	
Skeletal muscle actin 8 (*actinsk8*)	Myofibrillar contraction protein	F: CCCGCCACTTACGTTACCAT R: ACCTTCATAGACGGGGACCA	117	
Troponin C (*tnnc*)	Conformational changes on the thin filament	F: TCAGGAGGTCATCGCTGAGA R: GCCCTTGTCATAGATGCGGA	151	

**Table 2:** Selection output of the most stable candidate reference genes under different sample comparisons.

	FAS ^1^		MAS ^2^		FAA ^3^		MAA ^4^	
**FAS ^1^**			**0.301**	0.432	0.150	0.223	0.211	0.284
**MAS ^2^**	*eif*	*actb*			**0.341**	**0.238**	**0.416**	**0.404**
**FAA ^3^**	*eif*	*actb*	***actb***	***actb***			0.132	0.168
**MAA ^4^**	*eif*	*actb*	***actb***	***actb***	*eif*	*actb*		

Explanations: Below diagonal—genes identified by the two algorithms as the most stable for each comparison (e.g., FAS vs. MAS), above diagonal—identified by the two algorithms as the most stable for each comparison (e.g., FAS vs. MAS), shaded grey—genes/values indicated by the NormFinder (SD); white—genes/values indicated by the geNorm (M-Value); bolded—NormFinder and geNorm identified the same gene (the most stable). ^1^ FAS—female abdomen spring;.^2^ MAS—male abdomen spring; ^3^ FAA—female abdomen autumn; ^4^ MAA—male abdomen autumn.
